# PRRSV-Vaccinated, Seronegative Sows and Maternally Derived Antibodies (I): Impact on PRRSV-1 Challenge Outcomes in Piglets

**DOI:** 10.3390/vaccines11121745

**Published:** 2023-11-23

**Authors:** Jorian Fiers, Dominiek Maes, Ann-Brigitte Cay, Laurent Mostin, Anna Parys, Marylène Tignon

**Affiliations:** 1 Unit Viral Re-Emerging, Enzootic and Bee Diseases, Department Infectious Diseases in Animals, Sciensano, Groeselenbergstraat 99, 1180 Ukkel, Belgium; 2 Unit of Porcine Health Management, Department of Reproduction, Obstetrics and Herd Health, Faculty of Veterinary Medicine, Ghent University, Salisburylaan 133, 9820 Merelbeke, Belgium; dominiek.maes@ugent.be; 3 Unit Experimental Centre, Department Infectious Diseases in Animals, Sciensano, Kerklaan 68, 1830 Machelen, Belgium

**Keywords:** PRRSV, vaccination, viremia, challenge, immune response, maternally derived antibodies

## Abstract

Porcine Reproductive and Respiratory Syndrome Virus (PRRSV) remains an infectious agent with high importance in the swine industry. In this study, the influence of maternally derived antibodies (MDAs) on an experimental PRRSV-1 challenge is investigated. Piglets included in the study (*n* = 36) originated from a Belgian farrow-to-finish herd in which the sow population was routinely vaccinated with a modified live vaccine against PRRSV. Eighteen piglets were born from three PRRSV-seropositive sows (responders to vaccination) and had a clear presence of PRRSV-specific MDAs (E+ piglets). The other eighteen piglets were born from three PRRSV-seronegative sows (non-responders to vaccination) and did not have PRRSV-specific MDAs (E− piglets). In each group, twelve piglets were intranasally challenged with a high dose of the heterologous PRRSV-1 07V063 strain, the remaining piglets were mock-challenged (PBS) and served as controls. During the first days after infection, higher serum viremia and nasal shedding were observed in the challenged E− piglets compared to the challenged E+ piglets. However, at 10 days post-infection, the peak serum viremia was significantly higher in the E+ piglets in comparison to the E− piglets and serum viremia remained slightly higher in this group until the end of the study. Additionally, the two challenged groups had a different immune response to the PRRSV infection. The E− challenged piglets showed an earlier and more intense seroconversion, leading to significantly higher antibody titers at 10 dpi compared to the E+ challenged piglets. Furthermore, a trend towards both higher induction of serum IFN-γ and higher induction of IFN-γ secreting cells was observed in the E− challenged piglets. In contrast, a significantly higher induction of serum TNF-α at 7 dpi was seen in the E+ challenged piglets compared to the E− challenged piglets. The results gathered in this study suggest that PRRSV-specific MDAs induce partial protection during the early stages of infection but are not sufficient to protect against a high challenge dose. The presence of piglets lacking PRRSV-specific MDAs might pose a risk for PRRSV infection and enhanced transmission in pig farms in young piglets.

## 1. Introduction

Porcine Reproductive and Respiratory Syndrome (PRRS) is a swine disease, responsible for high production and financial losses worldwide. The first disease outbreaks, characterized by both reproductive losses and respiratory disease, were reported in the late 1980s in the United States and later, in 1990, in Europe [1,2,3]. In 1991, the pathogen responsible for the disease outbreaks was identified as a small RNA virus: the PRRS-virus (PRRSV) [4,5]. Nowadays, this virus is classified in two distinct species: *Betaarterivirus suid 1* (PRRSV-1) and *Betaarterivirus suid 2* (PRRSV-2), both belonging to the order *Nidovirales*, family *Arteriviridae* [6]. Three decades after the first reported PRRS outbreaks, the disease is still responsible for enormous production and economic losses worldwide [7,8]. These losses can be attributed to both the direct clinical effects of a PRRSV-infection and to the high incidence of secondary infections, since PRRSV-infected pigs have an impaired respiratory immune system [9,10]. Fortunately, there are several commercial PRRSV-vaccines available that can be administered to both sows and piglets, and that can aid in reducing the negative consequences of this disease. Both modified live vaccines (MLVs) and inactivated/killed vaccines (KV) are used in the field, with both types of vaccines having their specific (dis)advantages [11]. Importantly, the effectiveness of the used vaccines is strongly dependent on a combination of the used vaccine, the vaccine strategy, and the biosecurity practices on the farm [12].

The emergence of field reports stating the presence of ELISA seronegative sows (ELISA non-responders or anergic sows), despite repeated vaccination against PRRSV, is a concern that has remained understudied so far [13]. Recently, our research group has assessed the prevalence of these multiple PRRSV-vaccinated but ELISA seronegative sows in Belgium; a global prevalence of 3.5% to 4.1% of non-responding sows was found [14]. Similar observations of anergic sows after repeated PRRSV vaccination were reported at the same time by other research groups, with the global prevalence ranging from 1% to 4% [15,16]. A first consequence of this sow seronegative status for the progeny has been assessed in a follow-up study [17]. In short, piglets born from these PRRSV-vaccinated but seronegative sows lacked the presence of PRRSV-specific maternally-derived antibodies (MDAs). Due to this, they showed a stronger vaccine viremia and earlier seroconversion compared to piglets born from PRRSV-vaccinated, seropositive sows who had the presence of MDAs. In the context of piglet vaccination, this lack of MDAs might be considered beneficial, since it is known that PRRSV-specific MDAs can interfere with homologous piglet vaccination [18,19]. However, in the context of a PRRSV-infection, it could be hypothesized that piglets lacking MDAs might be less protected compared to piglets having the presence of MDAs. In the current study, the influence of MDAs on PRRSV-infection was investigated by experimentally challenging four-weeks-old piglets born from both PRRSV-vaccinated, seronegative, and PRRSV-vaccinated seropositive sows.

## 2. Materials and Methods

### 2.1. Study Design

**Figure 1:**
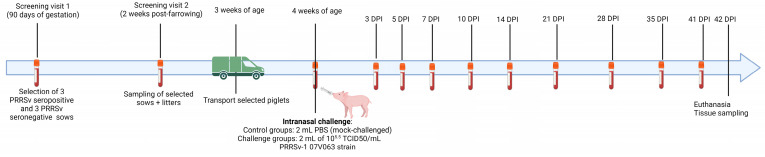
Schematic overview of the experimental challenge study. Created using Biorender.com.

The study design was approved by the Ethical Committee of Sciensano, with approval number 20221025-01. An overview of the study design can be found in Figure 1. The study contained thirty-six piglets (Hypor x German Piétrain) originating from a PRRSV-stable Belgian farrow-to-finish herd. The selected herd practiced a routine PRRSV vaccination of the sow population: intramuscular Porcilis MLV (MSD, Rahway, NJ, USA) vaccination at 60 days of gestation and at 6 days post-farrowing. A first on-herd screening visit was performed at 90 days of gestation, in which 49 sows of different parities were sampled for the analysis of PRRSV-specific antibodies. Based on this visit, three PRRSV-seropositive sows (responders to vaccination) and three PRRSV-seronegative sows (non-responders to vaccination) were selected. Two weeks post-farrowing, a second on-herd screening visit was performed in which both the selected sows and ten piglets born from each sow were blood sampled. Additionally, a follow-up visit was performed at 12 weeks post-farrowing to investigate whether the PRRSV-seronegative sows remained seronegative, despite having received an additional PRRSV vaccination (at 6 days post-farrowing). At three weeks post-farrowing, eighteen piglets originating from the three selected PRRSV-seropositive sows (E+ piglets; 6 piglets per sow) and eighteen piglets originating from the three selected PRRSV-seronegative sows (E− piglets; 6 piglets per sow) were transported to the experimental facilities of Sciensano (Machelen, Belgium). Upon arrival, the piglets were distributed in four experimental groups and six piglets were housed per pen: E+ controls (*n* = 6; pen 1), E− controls (*n* = 6; pen 2), E+ challenged (*n* = 12; pen 3 + pen 4), and E− challenged (*n* = 12; pen 5 + pen 6). After an acclimatization period of one week, the control groups were intranasally mock-challenged with 2 mL of PBS (1 mL per nostril), while the challenged groups were intranasally challenged with 2 mL containing 10^5.5^ tissue culture infectious dose with 50% end point per mL (TCID_50_/mL) of the PRRSV-1 07V063 strain (1 mL per nostril). All piglets were sampled for blood and nasal swabs at the moment of challenge (day 0) and at 3, 5, 7, 10, 14, 21, 28, 35, and 41 days post-infection (dpi). Rectal temperature was measured daily from day 0 until 16 dpi; a rectal temperature higher than 40 °C was considered as a fever. All piglets were euthanized by electrocution at 42 dpi and necropsied for the collection of right lung tissues (cranial lobe, middle lobe, and caudal lobe), bronchial lymph node, spleen, and tonsil of the soft palate. Unfortunately, one E+ challenged piglet died during blood sampling at 7 dpi; this piglet was removed from the study and was not included in any analysis.

### 2.2. Viruses

The PRRSV-1 07V063 strain (GenBank: GU737264), a Belgian field strain isolated in 2007, was provided by Dr. Hans Nauwynck (Ghent University, Merelbeke, Belgium) [20,21,22]. After receiving the viral stock, a three-passage propagation was performed in porcine alveolar macrophages (PAMs), which were cultured in RPMI 1640 + Glutamax (Gibco, Thermo Fisher Scientific, Waltham, MA, USA), supplemented with 10% fetal calf serum, 1% non-essential amino acids, 1% sodium pyruvate, 1% amphotericin B, 1% gentamycin, and 1% penicillin/streptomycin. The PRRSV-1 DV strain, which was derived from the Porcilis MLV, was propagated in MARC-145 cells cultured in minimal essential medium (MEM) (Gibco, Thermo Fisher Scientific, Waltham, MA, USA), supplemented with 0.1% amphotericin B, 0.1% gentamycin, and 0.5% penicillin/streptomycin. Viral titers were calculated by inoculating either PAMs or MARC-145 cells with ten-fold serial dilutions of the 07V063 strain or DV strain, respectively. Cells were incubated for 72 h at 37 °C, washed with PBS, and heat-fixed (30 min at 37 °C + 80 min at 80 °C). Finally, a PRRSV-specific monoclonal antibody was used to stain the fixed cells, as previously described [14]. The final titer (50% endpoint dilution) was determined using the Reed–Muench method [23].

### 2.3. Antibody Analysis

The absence or presence of PRRSV-specific antibodies (Abs) was determined using the commercially available IDEXX PRRS X3 Ab ELISA test (IDEXX Laboratories, Westbrook, ME, USA), which can be considered the golden standard for PRRSV Ab analysis [9]. Additionally, porcine circovirus type 2 (PCV2) specific Abs were analyzed using the Biochek PCV2 Antibody Test (Biochek, Reeuwijk, The Netherlands). Both the PRRSV ELISA and the PCV2 ELISA were performed as previously described [17]. Samples having a sample-to-positive (S/*p*) value ≥ 0.4 were considered as seropositive in the PRRSV ELISA. Samples with a S/*p* value ≥ 0.5 were considered as seropositive in the PCV2 ELISA.

### 2.4. Cytokine Analysis

The concentration of interferon-gamma (IFN-γ) and tumor necrosis factor-alpha (TNF-α) in serum was determined using the commercial IFN gamma Porcine ELISA kit (Invitrogen—Thermo Fisher Scientific, Waltham, MA, USA) and the commercial TNF alpha Porcine ELISA kit (Invitrogen—Thermo Fisher Scientific, Waltham, MA, USA), respectively. The manufacturers’ guidelines were followed for both tests.

### 2.5. Virological Analysis

RNA extraction was performed using the IndiMag Pathogen kit and IndiMag 48 s instrument (Indical Bioscience, Leipzig, Germany). During each RNA extraction, a four-point serial dilution (ten-fold dilutions) of the PRRSV-1 07V063 challenge strain was included. The presence of PRRSV was analyzed by RT-qPCR on the extracted RNA using the VetMAX PRRSV EU and NA 2.0 kit (Thermo Fisher Scientific, Waltham, MA, USA) on the QuantStudio 5 Real-Time PCR System (Thermo Fisher Scientific, Waltham, MA, USA). A relative quantification was performed based on the 07V063 serial dilutions that were included in each extraction and RT-qPCR run. First, the absolute concentration of the 07V063 stock was determined by droplet digital PCR (ddPCR) using the QX600 Droplet Digital PCR System (Bio-Rad Laboratories, Hercules, CA, USA) with the One-Step RT-ddPCR Advanced Kit for Probes (Bio-Rad Laboratories, Hercules, CA, USA) and 07V063 specific primers and probes (Integrated DNA Technologies, Coralville, IA, USA). Next, a standard curve was generated during each RT-qPCR run based on the obtained Ct-values for the serial dilutions of the 07V063 strain and the absolute concentration (Log copies/µL) obtained by ddPCR. Finally, the relative concentration of each sample included in one run was calculated by interpolating the obtained Ct-values on the standard curve generated during that run. Prior to extraction, nasal swabs were suspended in 1 mL of PBS using the Tissue Lyser Bloc (Qiagen, Hilden, Germany); tissue samples were cut in fine pieces, weighted, and suspended in 1 mL of PBS using the Tissue Lyser Bloc (Qiagen, Hilden, Germany). The obtained viral load of tissue samples was standardized to 1 g of tissue.

### 2.6. Interferon-Gamma ELISpot

The ELISpot Plus: Porcine IFN-γ (ALP) (Mabtech AB, Nacka Strand, Sweden) was used to quantify the number of IFN-γ secreting cells (IFN-γ SCs). First, the isolation of peripheral blood mononuclear cells (PBMCs) was performed on heparinized blood on the day of collection, using SepMate PBMC isolation tubes (STEMCELL Technologies, Vancouver, Canada) with Histopaque-1077 (Sigma-Aldrich, Saint-Louis, MI, USA) as a density gradient. Isolated PBMCs were counted using the LUNA-FL Dial Fluorescence Cell Counter (Logos Biosystems, Gyeonggi-do, Republic of Korea). PBMCs were plated at a density of 5 ∗ 10^5^ PBMCs per well (100 µL) and 100 µL of stimuli was added to each well. For each sample, the following stimulation conditions were included (in duplicate): 100 µL of 10^3.9^ TCID_50_/mL of the PRRSV-1 DV strain, 100 µL supernatant of mock-inoculated MARC-145 cells (cell-culture medium; negative control), and 100 µL of 1 mg/mL Phytohemagglutinin-M (PHA-M; positive control; Roche Holding AG, Basel, Switzerland). Cells and stimuli were incubated for 20–24 h at 37 °C and staining was performed according to the manufacturers’ guidelines. ELISpot plates were analyzed on the CTL Q6 Ultra-V Analyzer (ImmunoSpot, Cleveland, OH, USA). For each stimulation condition, the final number of IFN-γ SCs/5 ∗ 10^5^ PBMCs was calculated as the average number of spots counted on the two duplicates. If a clear discrepancy was observed between the two duplicates, the sample was removed from analysis. For the PRRSV-recall assay, the number of IFN-γ SCs/5 ∗ 10^5^ PBMCs in the PRRSV-1 DV stimulated condition was subtracted by the number of IFN-γ SCs/5 ∗ 10^5^ PBMCs in the negative control stimulated condition for each sample.

### 2.7. Statistics

Statistical analysis and visualization of all results was performed using GraphPad Prism version 9.4.1 (GraphPad Software, San Diego, CA, USA). The Shapiro–Wilk test was used to perform a normality check on all data prior to statistical comparison. Statistical differences in normally distributed data were calculated using either an unpaired *t*-test for comparison between two experimental groups or one-way ANOVA with Sidaks multiple comparisons test for comparisons between more than two experimental groups. In non-normal distributed data, statistical differences were calculated using either the Mann–Whitney Test for comparison between two experimental groups or the Kruskal–Wallis test with Dunn’s multiple comparisons test for comparisons between more than two experimental groups. All results are written as mean ± standard deviation. The results of statistical tests with *p*-values < 0.05 were considered to be statistically significant. On the figures, statistical significance is visualized with asterisks: * *p*-value < 0.05; ** *p*-value < 0.01; *** *p*-value< 0.005; **** *p*-value < 0.001.

## 3. Results

### 3.1. Sow and Piglet Selection

During the first screening visit, 44/49 (89.8%) sows tested PRRSV seropositive, while 5/49 (10.2%) sows tested PRRSV seronegative (Supplementary Figure S1A). Of these sows, three PRRSV-seronegatives and three PRRSV-seropositives were selected; these sows were resampled at 14 days post-farrowing (dpf) and 12 weeks post-farrowing (wpf) (Table 1). Interestingly, despite having received an additional PRRSV vaccination at 6 dpf, the PRRS-seronegative sows remained seronegative at 12 wpf. In contrast, an increase in S/*p* value was observed in the PRRSV seropositive sows due to the additional vaccination (Table 1).

**Figure 2:**
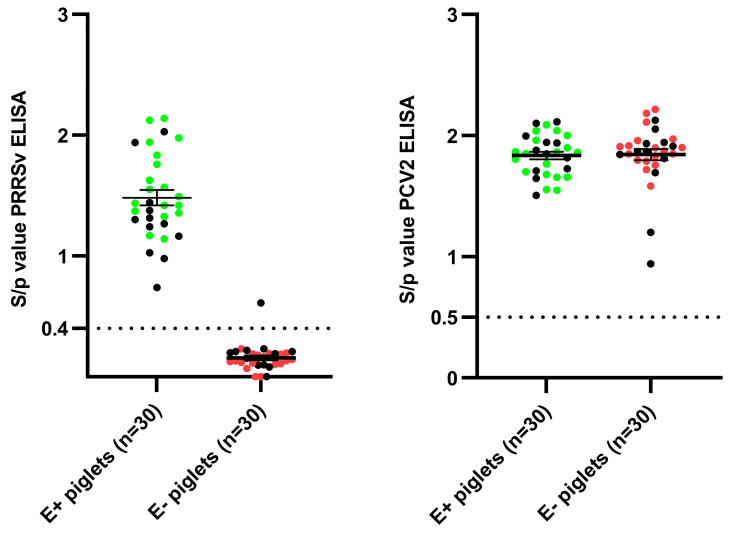
Presence of PRRSV-specific antibodies (**left**) and PCV2-specific antibodies (**right**) in thirty piglets originating from three PRRSV-vaccinated, seropositive sows (E+ piglets) and thirty piglets originating from three PRRSV-vaccinated, seronegative sows (E− piglets). Results are shown as dots representing the individual S/*p* values for each piglet. The cut-off value for seropositivity in each ELISA test is shown as a dotted line. Green dots represent the selected E+ piglets, while red dots represent the selected E− piglets. Error bars represent the mean S/*p*-value ± standard error of the mean (SEM).

Both the selected PRRSV-seropositive sows and the selected PRRSV-seronegative sows had a clear presence of PCV2 antibodies (Abs) at 14 dpf (Supplementary Figure S1B). Consequently, the presence of PCV2 MDAs in the progeny can be used as a control for colostrum intake [14]. Next to the selected sows, ten piglets originating from each selected sow were blood sampled during the second screening visit (14 dpf). All thirty piglets originating from the three PRRSV-seropositive sows tested PRRSV-seropositive, and thus, had received PRRSV-specific MDAs (Figure 2). In contrast, all piglets originating from the PRRSV-seronegative sows tested PRRSV-seronegative as well, with the exception of one piglet, born from sow 5, who had an S/*p* value just above the cut-off for seropositivity (Figure 2). The final selection of eighteen piglets born from the three PRRSV-seropositive sows (E+ piglets) and eighteen piglets born from the three PRRSV-seronegative sows (E− piglets) was based on the presence of both PRRSV-specific and PCV2-specific MDAs (control for colostrum intake) (Figure 2).

### 3.2. Rectal Temperature

**Figure 3:**
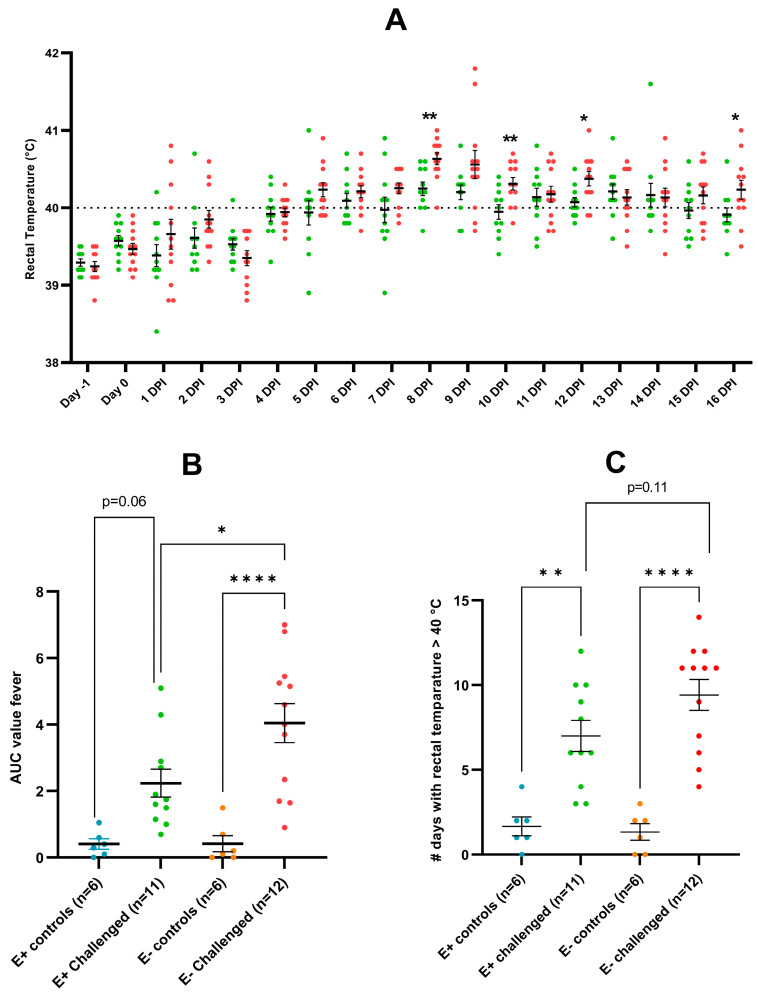
Analysis of rectal temperature in PRRSV-seropositive piglets (E+ piglets) and PRRSV-seronegative piglets (E− piglets) after either intranasal mock-challenge with PBS (control groups) or intranasal PRRSV-1 07V063 challenge (challenged groups). (**A**) Results are shown as the rectal temperature (°C) measured from 1 day before challenge (Day-1) until 16 days post-infection (dpi). Green dots represent the challenged E+ piglets (*n* = 11), while red dots represent the E− challenged piglets (*n* = 12). A dotted line represents the cut-off for fever. (**B**) Results are shown as the area under the curve (AUC) values for fever (rectal temperature > 40 °C); calculated from 1 dpi until 16 dpi. Individual dots represent the AUC value for each piglet, grouped per experimental group. Error bars represent the mean AUC value ± standard error of the mean (SEM) for each experimental group. (**C**) Results are shown as the number of days with fever (rectal temperature > 40 °C); calculated from 1 dpi until 16 dpi. Individual dots represent the number of days with fever for each piglet, grouped per experimental group. Error bars represent the mean number of fever days ± standard error of the mean (SEM) for each experimental group. On the figures, statistical significance is visualized with asterisks: * *p*-value < 0.05; ** *p*-value < 0.01; **** *p*-value < 0.001.

Post-challenge, an overall trend towards higher rectal temperature was observed in the E− challenged piglets compared to the E+ challenged piglets (Figure 3A). At 8, 10, 12, and 16 dpi, the rectal temperature was significantly higher in the E− challenged group compared to the E+ challenged group. Moreover, the mean area under the curve (AUC) value for fever (rectal temperature > 40 °C) was significantly higher in the E− challenged piglets (4.05 ± 2.03) compared to both the E+ challenged piglets (2.24 ± 1.40) (*p* = 0.0183) and the E− control piglets (0.42 ± 0.59) (*p* < 0.0001) (Figure 3B). Furthermore, the mean number of days with fever was significantly higher in the challenged groups compared to their respective control groups, while this was non-significantly higher (*p* = 0.11) in the E− challenged piglets (9.42 ± 3.18) compared to the E+ challenged piglets (7.00 ± 3.03) (Figure 3C).

### 3.3. Virological Analysis

All samples gathered during the on-herd screening visits (sows + piglets) as well as all piglets at day 0 tested negative in PCR for PRRSV. Additionally, all control piglets tested PCR negative during the entire study, confirming that there was no PRRSV infection in the transported piglets prior to the challenge experiment and there was no PRRSV contamination from the challenged pens towards the control pens.

**Figure 4:**
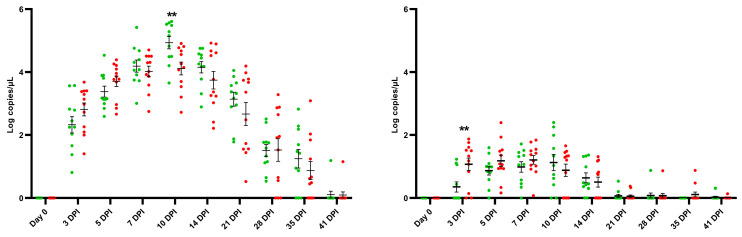
Viral load in the serum (**left**) and nasal swabs (**right**) of PRRSV-seropositive piglets (E+ piglets; *n* = 11) and PRRSV-seronegative piglets (E− piglets; *n* = 12), intranasally challenged with the PRRSV-1 07V063 strain at 4 weeks of age. Green dots represent the viral load in the E+ challenged piglets, while red dots represent the viral load in the E− challenged piglets. Viral load was analyzed until 41 days post-infection (dpi). Error bars represent the mean viral load ± standard error of the mean (SEM) calculated at each time point. ** *p*-value < 0.01.

During early infection (0–5 dpi), a trend towards a higher viral load in both the serum and the nasal swabs of the challenged E− piglets compared to the challenged E+ piglets was observed (Figure 4). At 3 dpi, a significantly higher viral load (*p* = 0.0085) was observed in the nasal swabs of the challenged E− piglets (1.07 ± 0.68 Log copies/µL) compared to the challenged E+ piglets (0.35 ± 0.52 Log copies/µL). At 7 dpi, the viral load in both the serum and the nasal swabs was similar between the two challenged groups. At 10 dpi, serum viral load reached its peak in both challenged groups. Interestingly, this peak was significantly higher (*p* = 0.0085) in the E+ challenged piglets (4.93 ± 0.65 Log copies/µL) compared to the E− challenged piglets (4.11 ± 0.70 Log copies/µL). This result was confirmed in an end-point dilution assay; the E+ challenged piglets (4.03 ± 0.54 Log TCID_50_/mL) had a significantly higher (*p* = 0.0007) serum viral titer at 10 dpi compared to the E− challenged piglets (3.11 ± 0.57 Log TCID_50_/mL) (Supplementary Figure S2). From 10 dpi until 21 dpi, a trend towards higher serum viral load was observed in the E+ challenged piglets. By the end of the study (41 dpi), all challenged piglets became PCR negative, with the exception of one E+ challenged piglet and one E− challenged piglet. In the nasal swabs, no additional differences in viral load between the two challenged groups were observed, and from 21 dpi onwards the viral load became lower than the limit of detection in almost all challenged piglets. Lastly, no significant differences were observed in the AUC value for viral load in the serum of the E+ challenged piglets (104.0 ± 12.39) compared to the E− challenged piglets (94.66 ± 28.03) nor for the AUC value for viral load in the nasal swabs between the E+ challenged piglets (13.58 ± 7.06) and the E− challenged piglets (15.54 ± 5.68).

**Figure 5:**
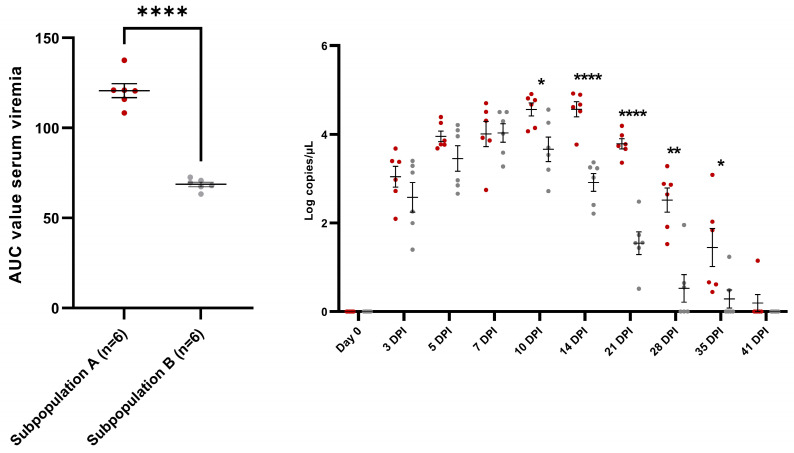
Viral load in the serum of twelve PRRSV-seronegative piglets (E− piglets), intranasally challenged with the PRRSV-1 07V063 strain at 4 weeks of age. Analysis of the AUC value for viral load in serum (**left**) showed a clear presence of two subpopulations: Subpopulation A (dark red dots) contains six E− piglets with a high AUC value for viral load in serum, while subpopulation B (grey dots) contains six E− piglets with a low AUC value for viral load in serum. The evolution of serum viral load (**right**) is presented as dark red dots for the E− piglets of subpopulation A and grey dots for the E− piglets of subpopulation B. Error bars represent the mean viral load ± standard error of the mean (SEM) calculated for each time point. * *p*-value < 0.05; ** *p*-value < 0.01; **** *p*-value < 0.001.

Interestingly, two subpopulations were observed in the E− challenged piglets: subpopulation A contained six piglets with an overall high AUC value for viral load in serum (120.7 ± 9.62) which was significantly higher (*p* < 0.0001) than the overall low AUC-value (68.63 ± 3.20) seen in subpopulation B (Figure 5). Furthermore, a clear sow effect was observed in these subpopulations: the four siblings originating from sow 4 all belonged to subpopulation A, while three out of four siblings originating from both sow 5 and sow 6 belonged to subpopulation B (Table 1). An additional analysis was performed comparing the evolution of serum viral load in these two subpopulations (Figure 5). During early infection (0–5 dpi), a non-significant trend towards higher viral load in serum was observed in subpopulation A. At 7 dpi, no differences were observed between both subpopulations, however, at this point subpopulation B already reached its peak viral load. From 10 dpi until 35 dpi, the viral load in serum remained significantly higher in subpopulation A compared to subpopulation B.

**Figure 6:**
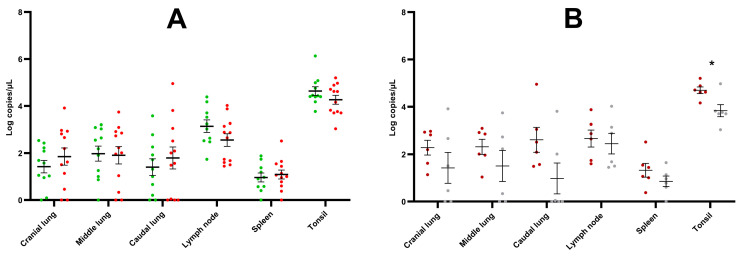
(**A**) Viral load in the tissues of PRRSV-seropositive piglets (E+ piglets; *n* = 11) and PRRSV-seronegative piglets (E− piglets; *n* = 12) 42 days after intranasal challenge with the PRRSV-1 07V063 strain. Green dots represent the viral load in the E+ piglets, while red dots represent the viral load in the E− piglets. (**B**) Viral load in the tissues of two subpopulations of E− challenged piglets 42 days after intranasal challenge with the PRRSV-1 07V063 strain. Subpopulation A (dark red dots) contains six E− piglets with a high AUC value for viral load in serum, while subpopulation B (grey dots) contains six E− piglets with a low AUC value for viral load in serum. Error bars represent the mean viral load ± standard error of the mean (SEM) calculated for each time point. * *p*-value < 0.05.

At 42 dpi, all piglets were euthanized and necropsied. Right lung tissues (cranial lobe, middle lobe and caudal lobe), bronchial lymph node, spleen, and tonsils of the soft palate were collected. Overall, the viral load was highest in the tonsil, followed by the lymph node, the lung tissues, and the spleen (Figure 6A). No significant differences were observed in the viral load of the tissues of the E+ challenged piglets compared to the E− challenged piglets. An additional analysis was performed, comparing the viral load in the tissues of the two subpopulations of E− challenged piglets (Figure 6B). Overall, a non-significant trend towards higher viral load was observed in the lung tissues, lymph nodes, and spleens of subpopulation A. In the tonsils, a significantly higher (*p* = 0.014) viral load was observed in subpopulation A (4.70 ± 0.34) compared to subpopulation B (3.83 ± 0.63).

### 3.4. Antibody Response

**Figure 7:**
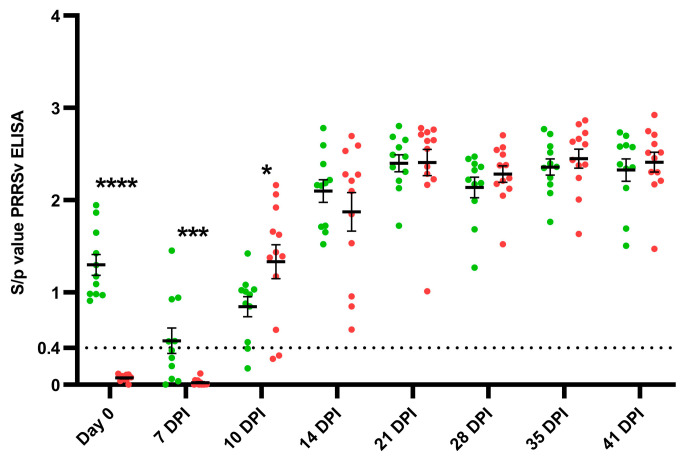
Evolution of PRRSV-specific antibodies in PRRSV-seropositive piglets (E+ piglets; *n* = 11) and PRRSV-seronegative piglets (E− piglets; *n* = 12) after the intranasal challenge with the PRRSV-1 07V063 strain at 4 weeks of age. ELISA sample-to-positive (S/*p*) values are shown as dots for each piglet (E+ piglets: green dots, E− piglets: red dots) from the day of challenge (day 0) until 41 days post-infection (dpi). A dotted line shows the cut-off value for seropositivity. Error bars represent the mean S/*p*-value ± standard error of the mean (SEM) for each experimental group at each time point. * *p*-value < 0.05; *** *p*-value< 0.005; **** *p*-value < 0.001.

A first induction of PRRSV-specific antibodies in the challenged piglets was observed between 7 dpi and 10 dpi (Figure 7). The E− challenged piglets showed a higher early antibody response, with a significantly higher (*p* = 0.037) ELISA S/*p* value (1.33 ± 0.064) compared to the E+ challenged piglets (0.85 ± 0.36) at 10 dpi. From 14 dpi until the end of the study, no differences in induced antibodies were observed between both challenged groups. A more rapid waning of the MDAs was seen in the E+ challenged piglets compared to the E+ control piglets: at 7 dpi only 5/11 (54.6%) E+ challenged piglets were seropositive, while all six E+ control piglets were still seropositive at that time point (Supplementary Figure S3). The E− control piglets remained PRRSV seronegative throughout the study.

### 3.5. Cytokine Induction in Serum

In general, a limited induction of IFN-γ was observed in the serum of the challenged piglets (Supplementary Figure S4). At 3 dpi, a significantly higher (*p* = 0.021) IFN-γ induction was observed in the serum of the E− challenged piglets (26.23 ± 15.87 pg/mL) compared to the E+ challenged piglets (8.48 ± 11.37). At 10 dpi, a significantly higher (*p* = 0.038) IFN-γ induction was observed in the E− challenged piglets (10.93 ± 13.91 pg/mL) compared to the E− controls (0.0 ± 0.0 pg/mL).

**Figure 8:**
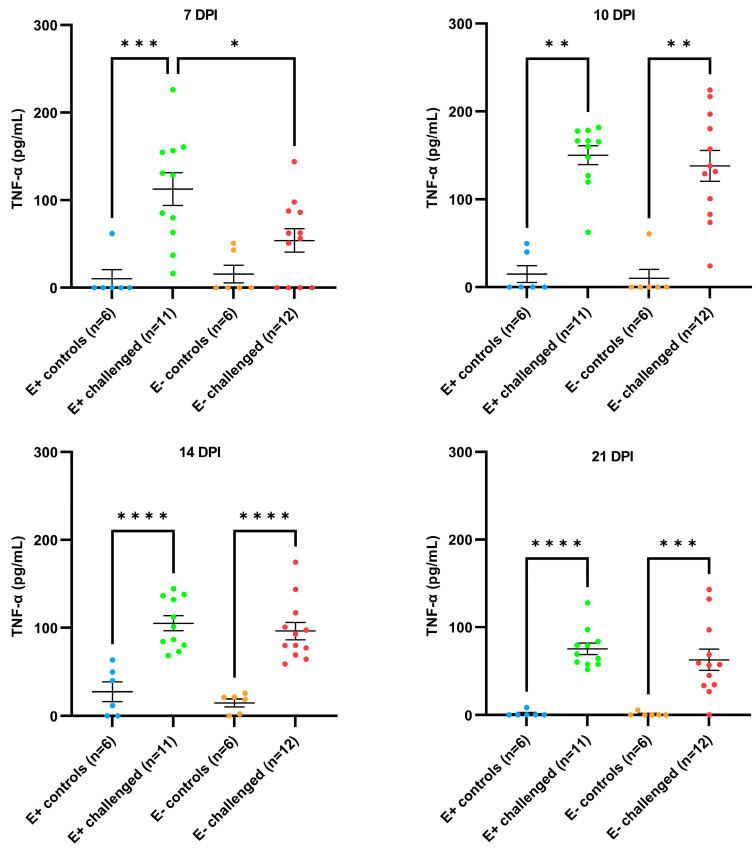
Evolution of TNF-α concentration in the serum of PRRSV-seropositive piglets (E+ piglets) and PRRSV-seronegative piglets (E− piglets) after either intranasal mock-challenge with PBS (control groups) or intranasal PRRSV-1 07V063 challenge (challenged groups). Individual dots represent the TNF-α concentration of each piglet at 7 days post-infection (dpi), 10 dpi, 14 dpi, and 21 dpi. Error bars represent the mean TNF-α ± standard error of the mean (SEM) for each experimental group at each time point. * *p*-value < 0.05; ** *p*-value < 0.01; *** *p*-value< 0.005; **** *p*-value < 0.001.

In contrast to the IFN-γ induction, a clear induction of TNF-α was observed in the serum of the challenged piglets from 7 dpi until 21 dpi (Figure 8). At 7 dpi, a significantly higher TNF-α induction was observed in the serum of the E+ challenged piglets (112.6 ± 62.18 pg/mL) compared to the E+ control piglets (10.32 ± 25.27) (*p* = 0.0005) and the E− challenged piglets (53.99 ± 46.71) (*p* = 0.017). At 10 dpi, 14 dpi, and 21 dpi, a significantly higher TNF-α induction was observed in the serum of both E+ challenged piglets and E− challenged piglets compared to their respective controls; no differences were observed between both challenged groups.

### 3.6. IFN-γ Secreting Cells

**Figure 9:**
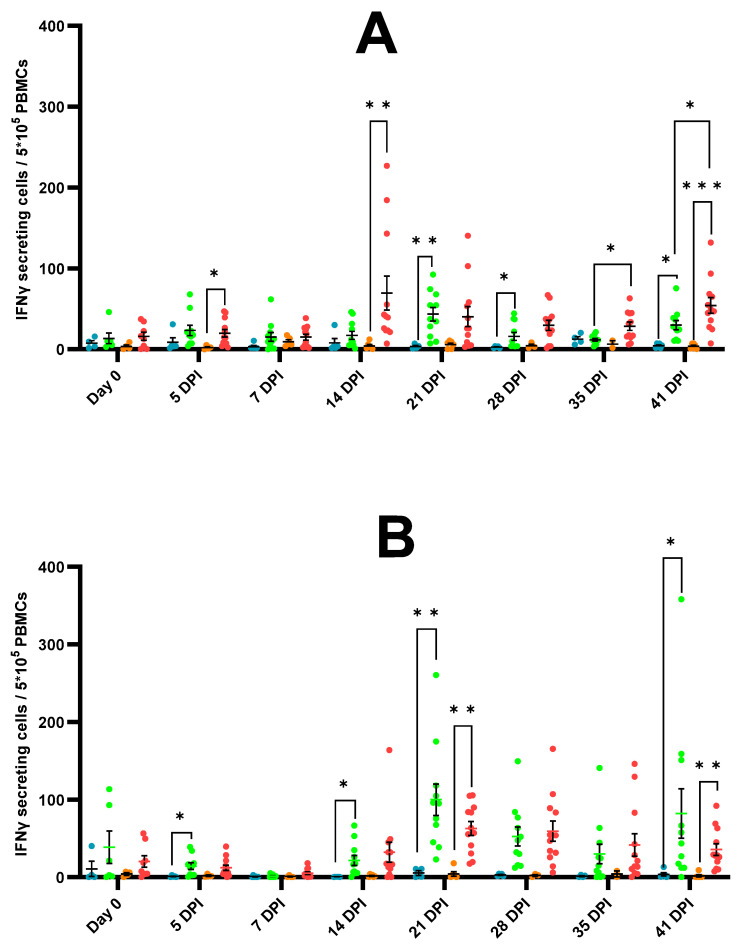
Presence of interferon-γ secreting cells (IFN-γ SCs) in PRRSV-seropositive piglets (E+ piglets) and PRRSV-seronegative piglets (E− piglets) after either intranasal mock-challenge with PBS (control groups) or intranasal PRRSV-1 07V063 challenge (challenged groups). Individual dots represent the number of IFN-γ SCs for each piglet per group (blue dots = E+ controls, green dots = E+ challenged, orange dots = E− controls, red dots = E− challenged). Error bars represent the mean number of IFN-γ SCs ± standard error of the mean (SEM) for each experimental group at each time point. (**A**) Number of IFN-γ SCs in non-stimulated conditions (cell-culture medium). (**B**) Number of IFN-γ SCs in a PRRSV-1 recall assay (Number of IFN-γ SCs after PRRSV-1 stimulation—Number of IFN-γ SCs after cell-culture medium stimulation). * *p*-value < 0.05; ** *p*-value < 0.01; *** *p*-value< 0.005.

In non-stimulated conditions (cell-culture medium), an overall trend towards a higher induction of IFN-γ secreting cells (IFN-γ SCs) was observed in the challenged piglets compared to their respective controls and in the E− challenged piglets compared to the E+ challenged piglets (Figure 9A). A significantly higher number of IFN-γ SCs was observed in the E− challenged piglets compared to the E+ challenged piglets at 35 dpi and 41 dpi. The relatively high observed induction of IFN-γ SCs in non-stimulated conditions interfered with the interpretation of the PRRSV-1 recall assay. However, a PRRSV-1 specific induction of IFN-γ SCs was still observed in the challenged piglets, while this induction was not observed in the control piglets (Figure 9B).

## 4. Discussion

In this study, the impact of the sow immune status and the consequent presence or absence of maternally derived antibodies (MDAs) on PRRSV-1 challenge outcomes in four-weeks-old piglets was analyzed. The trial contained two experimental challenge groups: piglets born from PRRSV-vaccinated, seropositive sows (E+ piglets; *n* = 11) and piglets born from PRRSV-vaccinated, seronegative sows (E− piglets; *n* = 12). All piglets in both groups had an adequate colostrum intake, as evidenced by the presence of PCV2-specific MDAs. All E+ piglets had the presence of PRRSV-specific MDAs, while these were absent in the E− piglets. Additionally, both E+ piglets and E− piglets originated from the same herd, ensuring that the observed challenge outcomes were only due to the presence or absence of MDAs or to possible genetic factors.

The lack of MDAs in the E− piglets led to an overall higher viral load in the first days post-challenge, both in serum and nasal swabs. Additionally, the E− piglets had a higher IFN-γ induction, a more intense early seroconversion and an overall higher induction of fever compared to the E+ piglets. In contrast, the E+ piglets had a higher peak viremia in serum (at 10 days post-challenge) and an earlier induction of serum TNF-α. Interestingly, there was a clear presence of two subpopulations within the E− piglets, with one subpopulation having a significantly lower overall viral load in serum due to a more rapid viral clearance. It can be hypothesized that this difference is due to certain genetic factor(s), which warrants further investigation. Several studies have stated the association between certain genetic regions and/or single nucleotide polymorphisms (SNPs) and PRRSV challenge outcomes [24,25,26]. It can be reasoned that these genetic factors might also play a role in the observed E− subpopulations in this study. To our knowledge, this is the first study in which the PRRSV challenge outcomes are compared between piglets with or without MDAs originating from the same commercial pig herd.

In previous studies, our research group showed that in 40% of the Belgian sow herds there was at least one PRRSV-vaccinated, but seronegative sow was present in twenty sows sampled [14]. Additionally, these PRRSV-vaccinated, seronegative sows gave rise to piglets without PRRSV-specific MDAs [17]. In this study, the consequence of this peculiar sow immune status on the infection susceptibility of the progeny was further elucidated. When compared to other PRRSV-challenge studies, the piglets born from the seronegative sows reacted to the challenge in a similar way as naïve piglets [19,20,21,22]. On the one hand, piglets born from the PRRSV-vaccinated, seronegative sows showed a stronger early viremia and nasal shedding compared to piglets born from the PRRSV-vaccinated, seropositive sows. It could be hypothesized that this population of piglets without PRRSV-specific MDAs poses an increased risk during the early stages of a possible wild-type PRRSV-introduction, as they might act as first reservoir for PRRSV-replication and transmission. On the other hand, piglets born from the PRRSV-vaccinated, seronegative sows showed a stronger early immune response with, consequently, a lower peak viremia compared to the piglets originating from the seropositive sows. The MDAs present in the latter group might have caused a first partial protection and/or shielding against the viral particles, leading to a reduced activation of the early immune response. Due to this, the piglets born from the PRRSV-vaccinated seropositive sows might have a delayed viral clearance, which could partially explain the observed higher peak viremia in serum. However, the observed difference in peak viremia has to be nuanced due to the presence of a subpopulation in the piglets born from the PRRSV-seronegative sows, which showed an overall low viremia and rapid viral clearance. A strong sow effect was observed in this subpopulation, which further enforces the hypothesis that certain sow genetic factors might play a role in the challenge outcomes of these piglets. Additionally, it could be reasoned that this might also explain why the sow itself did not respond to the routine PRRSV vaccination.

In the current study, the challenge outcomes in the two groups of piglets (with or without MDAs) were investigated in the absence of piglet vaccination. The question remains as to how these two groups of piglets will respond to a PRRSV-challenge after vaccination. It can be reasoned that the vaccine efficacy might be higher in the E− piglets due to the absence of MDA interference [17,18,19]. However, it has been shown that MLV vaccination does lead to a good protection against heterologous challenge, even in the presence of MDAs [27,28]. Additionally, the possible genetic factors in the piglets born from the seronegative sows might also influence the vaccine efficacy.

## 5. Conclusions

In conclusion, this study provided a first indication of the relevance of the mixed piglet population (piglets with and without PRRSV-specific MDAs), which is observed in at least 40% of the PRRSV-vaccinating sow herds in Belgium. This mixed piglet population is the direct consequence of a small proportion of sows that do not seem to react to routine PRRSV vaccination (absence of antibodies), since these sows do not transfer PRRSV-specific MDAs to their offspring. In this study, it was shown that piglets born from these sows have increased viral replication and nasal shedding in the first days post-challenge. This suggests that these piglets might be less protected against a wild-type infection during their first weeks of life and in this way, they might pose a risk for enhanced PRRSV-infection and transmission during early PRRSV-infection. Furthermore, it can be reasoned that these piglets might play a substantial role in the virus circulation within a PRRSV positive farm.

## Figures and Tables

**Table 1:** Evolution of antibodies (Abs) in the selected PRRSV-vaccinated sows and the presence of maternally derived Abs in the selected piglets originating from each sow. The presence of Abs was assessed using a commercially available PRRS ELISA test, with results shown as S/*p* values. Samples with an S/*p* value ≥ 0.4 are considered to be seropositive for PRRSV. Sows were intramuscular PRRSV-vaccinated at 60 days of gestation and 6 dpf; sow sampling was performed at 90 days of gestation, 14 dpf and 12 wpf; piglet sampling was performed at 14 dpf.

Sow Number	Parity	S/*p* Value (90 Days Gestation)	S/*p* Value (14 dpf)	S/*p* Value (12 wpf)	S/*p* Value Selected Piglets (14 dpf) (Mean ± SD)
Sow 1	5	2.06	1.54	1.73	1.52 ± 0.14
Sow 2	7	1.85	1.91	removed ^1^	1.94 ± 0.19
Sow 3	7	1.39	1.20	1.44	1.31 ± 0.13
Sow 4	6	0.26	0.19	0.29	0.12 ± 0.09
Sow 5	6	0.24	0.18	0.24	0.11 ± 0.02
Sow 6	2	0.36	0.39	0.25	0.17 ± 0.04

^1^ Sow 2 was removed from the herd after farrowing due to lameness.

## Data Availability

The data presented in this study are available on request from the corresponding author. The data are not publicly available due to privacy reasons.

## Supplementary Materials

The following supporting information can be downloaded at: https://www.mdpi.com/article/10.3390/vaccines11121745/s1, Figure S1A: Presence of PRRSV-specific antibodies in forty-nine PRRSV-vaccinated sows, sampled at 90 days of gestation (one month after the last PRRSV MLV vaccination); Figure S1B: Presence of PCV2-specific antibodies in the three selected PRRSV-seropositive sows and the three selected PRRSV-seronegative sows; Figure S2: Viral load in the serum of PRRSV-seropositive piglets (E+ piglets; *n* = 11) and PRRSV-seronegative piglets (E− piglets; *n* = 12), ten days after intranasal challenge (challenge at 4 weeks of age) with the PRRSV-1 07V063 strain; Figure S3: Evolution of PRRSV-specific antibodies in PRRSV-seropositive control piglets (E+ piglets; *n* = 6) and PRRSV-seronegative control piglets (E− piglets; *n* = 6) after intranasal mock-challenge with PBS at 4 weeks of age. Figure S4: Evolution of IFN-γ concentration in the serum of PRRSV-seropositive piglets (E+ piglets) and PRRSV-seronegative piglets (E− piglets) after either intranasal mock-challenge with PBS (control groups) or intranasal PRRSV-1 07V063 challenge (challenged groups).
