# Supplementary material for: PRRSV-Vaccinated, Seronegative Sows and Maternally Derived Antibodies (I): Impact on PRRSV-1 Challenge Outcomes in Piglets
Source: Vaccines (Basel). 2023 Nov 23;11(12):1745. doi: 10.3390/vaccines11121745 (PMC10748110; doi:10.3390/vaccines11121745)
Supplement: Supplementary file 1 [file vaccines-11-01745-s001.zip › vaccines-2725821-supplementary.pdf]

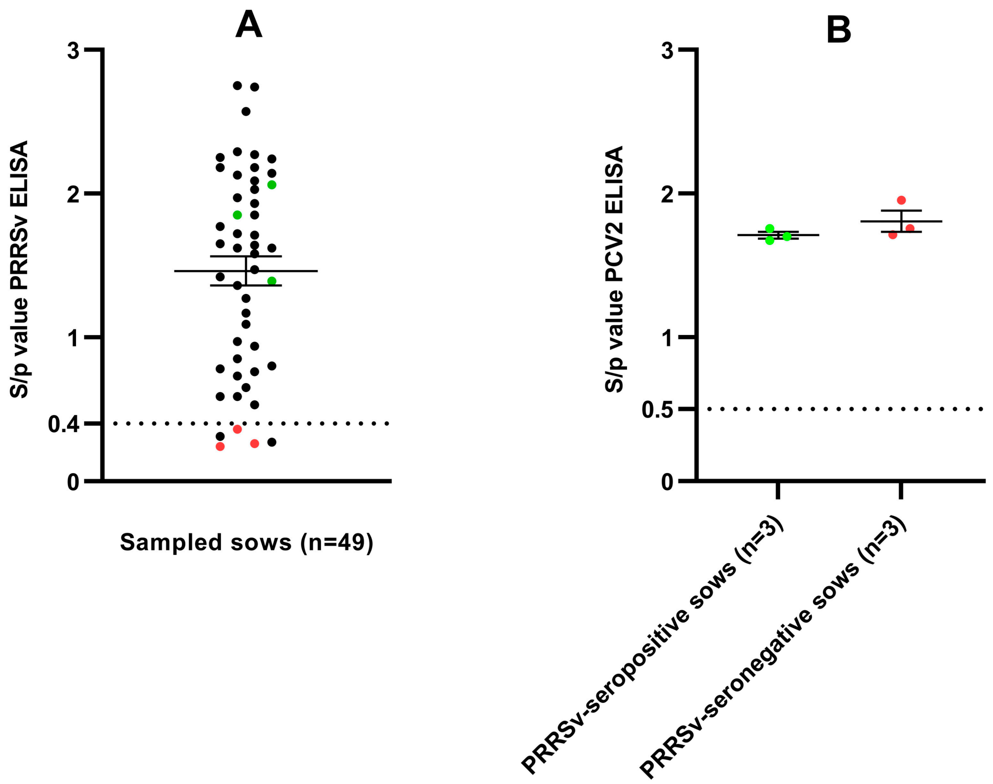

Supplementary Figure S1A. Presence of PRRSV-specific antibodies in forty-nine PRRSV-vaccinated sows, sampled at 90 days of gestation (one month after the last PRRSV MLV vaccination). Sample-to-positive values (S/p values) for each sow are presented as dots. The three selected PRRSV-seropositive sows are indicated with green dots ; the three selected PRRSV-seronegative sows are indicated with red dots. **Supplementary Figure S1B.** Presence of PCV2-specific antibodies in the three selected PRRSV-seropositive sows and the three selected PRRSV-seronegative sows. The cut-off value for seropositivity in each ELISA test is shown as a dotted line. Error bars represent the mean S/p-value ± standard error of the mean (SEM).

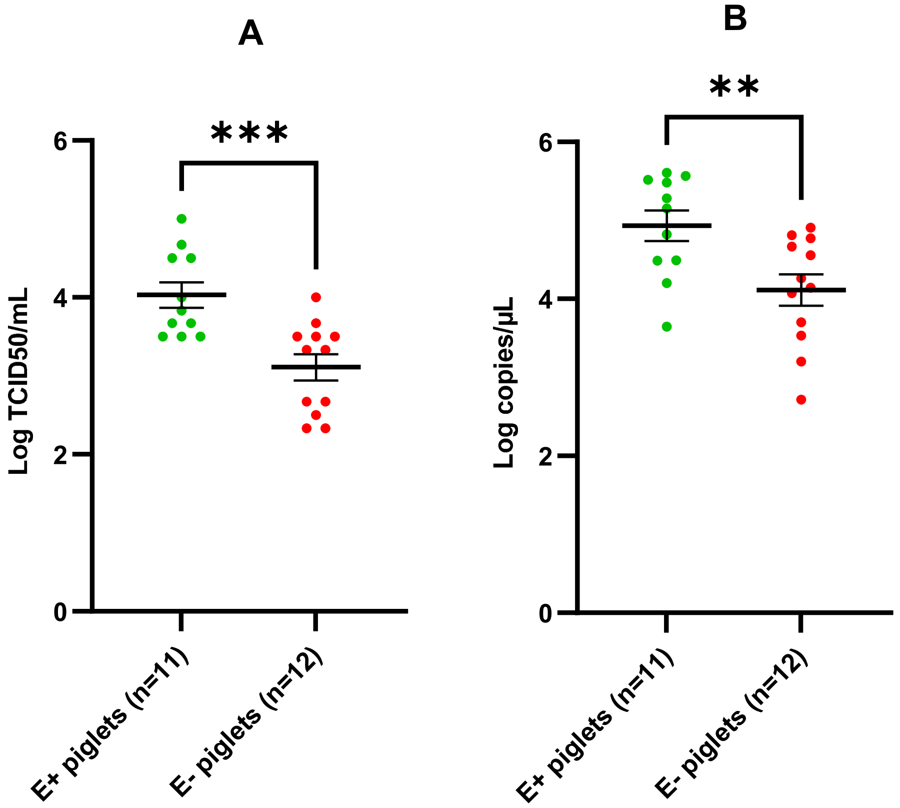

Supplementary Figure S2. Viral load in the serum of PRRSV-seropositive piglets (E+ piglets ; n=11) and PRRSV-seronegative piglets (E− piglets ; n=12), ten days after intranasal challenge (challenge at 4 weeks of age) with the PRRSV-1 07V063 strain. Green dots represent the viral load in the E+ challenged piglets, while red dots represent the viral load in the E− challenged piglets. **Supplementary Figure S2A.** Viral titer was quantified using an end point dilution assay on porcine alveolar macrophages. Results are shown as the tissue culture infectious dose with 50% end point / mL ($TCID_{50}$/mL) for each sample. **Supplementary Figure S2B.** Viral load was quantified using RT-qPCR. Error bars represent the mean viral load ± standard error of the mean (SEM).

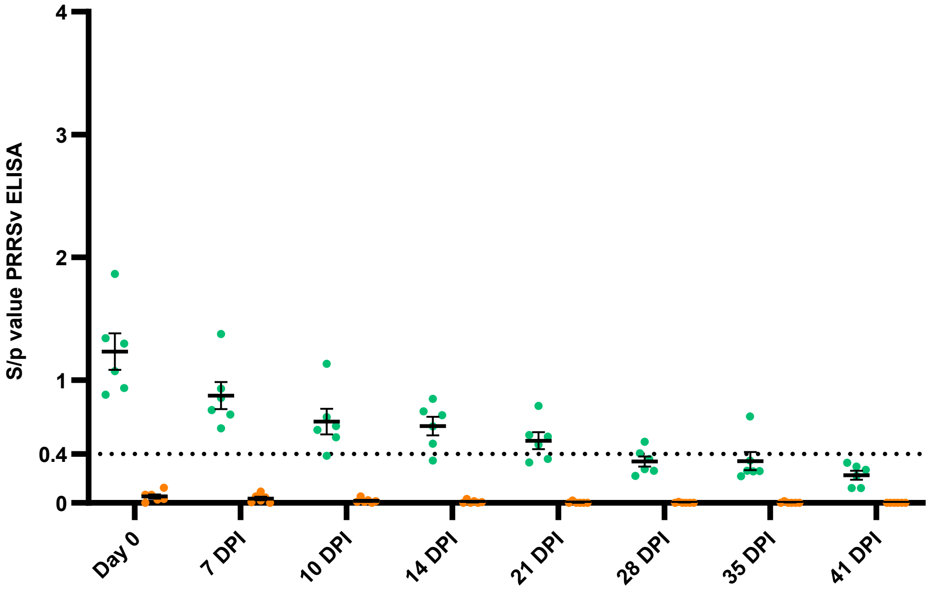

Supplementary Figure S3. Evolution of PRRSV-specific antibodies in PRRSV-seropositive control piglets (E+ piglets ; n=6) and PRRSV-seronegative control piglets (E− piglets ; n=6) after intranasal mock-challenge with PBS at 4 weeks of age. ELISA sample-to-positive (S/p) values are shown as dots for each piglet (E+ piglets: blue-green dots, E−piglets: orange dots) from day of challenge (day 0) until 41 days post-infection (dpi). A dotted line shows the cut-off value for seropositivity. Error bars represent the mean S/p-value ± standard error of the mean (SEM) for each experimental group at each time point.

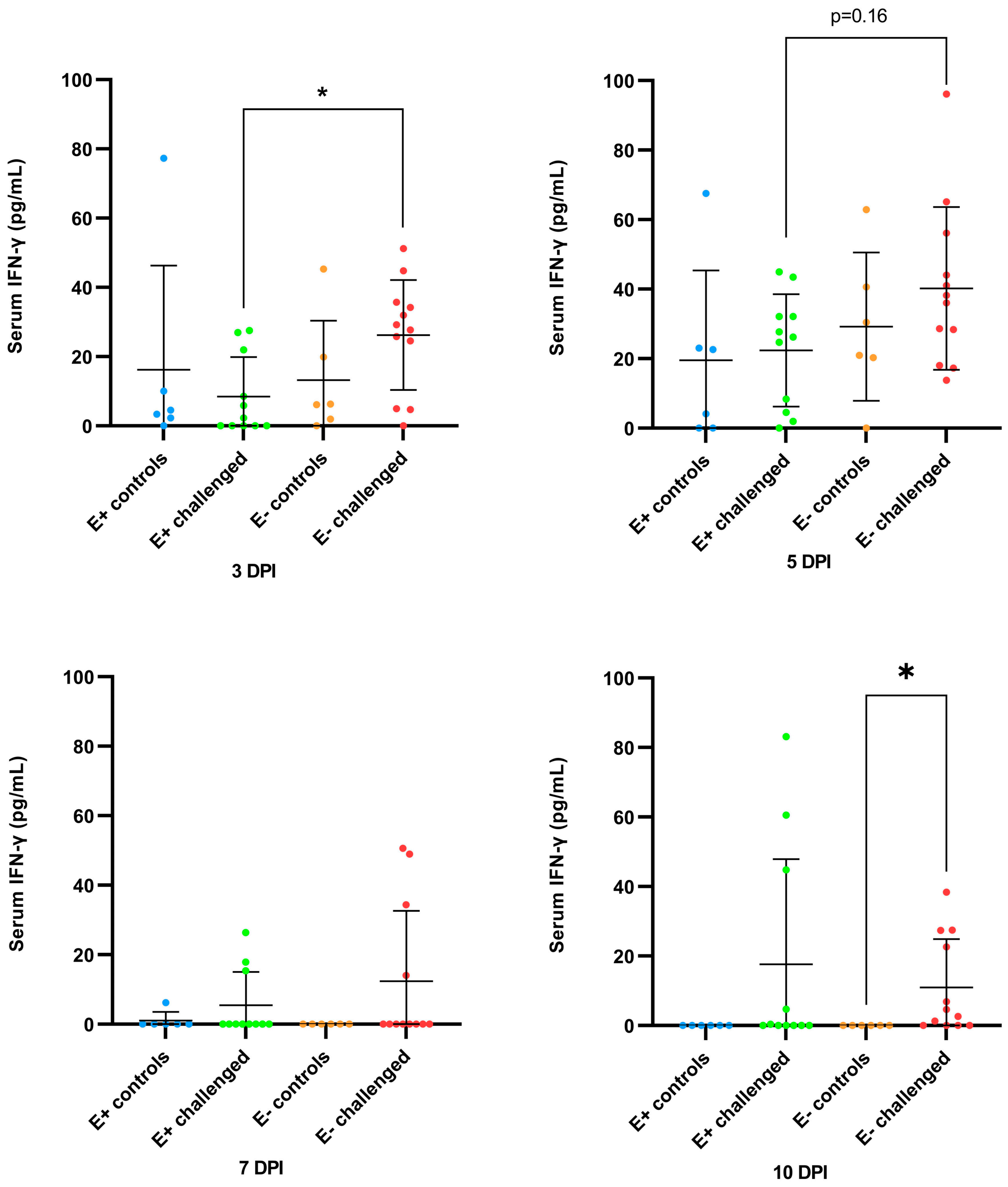

Supplementary Figure S4. Evolution of IFN-γ concentration in the serum of PRRSV-seropositive piglets (E+ piglets) and PRRSV-seronegative piglets (E− piglets) after either intranasal mock-challenge with PBS (control groups) or intranasal PRRSV-1 07V063 challenge (challenged groups). Individual dots represent the IFN-γ concentration of each piglet at 7 days post-infection (dpi), 10 dpi, 14 dpi and 21 dpi. Error bars represent the mean IFN-γ ± standard error of the mean (SEM) for each experimental group at each time point.
